# Attitudes, subjective norms and perceived behavioural control factors influencing Canadian secondary school students’ milk and milk alternatives consumption

**DOI:** 10.1017/S1368980024000661

**Published:** 2024-03-08

**Authors:** Carise M Thompson, Susan J Elliott, Samantha Meyer, Scott T Leatherdale, Shannon E Majowicz

**Affiliations:** University of Waterloo, 200 University Ave W, Waterloo, ON, Canada

**Keywords:** Adolescent, Milk, Theory of planned behaviour, Attitudes, Subjective norms

## Abstract

**Objective:**

The research objectives were to evaluate factors that influence Canadian secondary school students’ milk and milk alternatives (MMA) consumption and to explore associations through age and gender lenses.

**Design:**

A qualitative design was used, consisting of semi-structured interviews and photo-elicitation methods. Analysis was guided by the Theory of Planned Behaviour (TPB). Deductive and inductive thematic analyses were used to generate themes, charting data based on attributes such as gender and age.

**Setting:**

Interviews were held virtually or via telephone.

**Participants:**

Participants were twenty-eight high school students from Ontario, Canada, diverse in terms of gender and age.

**Results:**

Both desirable and undesirable beliefs about the health outcomes of consuming MMA were commonly discussed. These included health benefits such as strong bones, muscular strength, and growth, and health consequences like unwanted skin conditions, weight gain, and diseases. While boys and girls associated MMA consumption with muscular strength, boys predominantly considered this favourable, while girls discussed outcomes like unwanted skin conditions and weight gain more often. Adolescents’ perspectives on taste/perceived enjoyment, environmentally friendly choices and animal welfare also influenced their MMA preferences. Parental influences were most cited among social factors, which appeared to be stronger during early adolescence. Factors involving cost, time and accessibility affected adolescents’ beliefs about how difficult it was to consume MMA.

**Conclusions:**

Recommendations for shifting attitudes towards MMA are provided to address unfavourable beliefs towards these products. Interventions to increase MMA consumption among adolescents should include parents and address cost barriers.

Overall, Canadians’ milk and milk alternatives (MMA) and Ca consumption is low, and adolescents and girls are particularly at risk of inadequate intake^(1–5)^. MMA, including fluid milk, milk products and fortified plant-based alternatives, account for more than 38 % of dietary Ca consumed by Canadians and have been recommended as part of a healthy diet^(4,6)^. Yet, fewer Canadian adolescents aged 13–18 years met MMA recommendations in 2015 compared with 2004, while the prevalence of Ca inadequacy increased alarmingly to 86 % among females and 66 % among males of the same age^(4,5)^. While similar trends have been observed among younger children, children aged 1–8 years were 24–44 % more likely to meet their Ca requirements^(4)^ and children aged 6–12 years were 11 % more likely to meet MMA recommendations compared with adolescents aged 13–18 years^(5)^. Additionally, recent evidence shows that the prevalence of meeting recommendations for MMA begins to decline in childhood^(5)^ and drops incrementally during adolescence with increasing age^(1)^. One potential explanation for this is that adolescents are at an age of transition, shifting from being dependent on parents for choosing the types of foods they consume to making these decisions for themselves. These trends suggest adolescents represent a key demographic to understanding inadequate Ca and MMA intake. Besides age disparities, some research suggests that gender disparities are also associated with MMA consumption among adolescents^(1–3)^. Among Canadian adolescents, disproportionately more girls were not consuming recommended servings of MMA compared with boys^(1–3)^. Adolescent boys also consumed more servings of MMA per d compared with girls (2·36 *v*. 1·73)^(1)^ and had a higher frequency of milk consumption per d (1·48 *v*. 1·11 times per d) compared with adolescent girls^(2)^. Research to understand potential explanatory factors for the observed age and gender disparities in MMA and Ca intake is limited, particularly within the Canadian context.

Meeting Health Canada’s recommendations for Ca, which can be modified through MMA consumption, is associated with optimal bone health during adolescence^(7)^. In addition, establishing good bone health during adolescence is an important determinant of lifelong skeletal health, as approximately 90 % of the peak bone mass is reached by the age of 18 years^(7)^. Moreover, long-term consequences such as increased fracture risk are associated with inadequate Ca consumption during adolescence^(8)^. In addition to Ca, milk products provide an excellent source of protein, which is also important during development and has been recommended as such in the 2019 revision of Canada’s Food Guide^(9,10)^.

Previous research has associated demographic and behavioural characteristics with MMA and Ca consumption^(1,11–14)^. Among adolescents, factors such as ethnicity, activity level, weekly spending money and meeting Canada’s Food Guide recommendations for other food groups have been associated with meeting MMA guidelines^(1)^. Various modifiable factors have been associated with Ca and dairy consumption among other age groups, such as knowledge, outcome expectations related to health and immediate other benefits, taste, confidence in the product, perceptions about animal welfare, and beliefs about hormones or antibiotics^(11–15)^. Additional facilitators and barriers to Ca and dairy consumption have been identified among other age groups, including convenience, reinforcement, cost, habits, cultural values, favourable food and drink combinations, skipping meals, and availability^(11–15)^. Subjective norms related to family, friends and health professionals have similarly been associated with these behaviours among other age groups^(11–14)^. Research identifying barriers and facilitators to Canadians’ dairy consumption and Ca intake has mostly focused on adults^(11–13)^, although Racey *et al.* explored factors among Canadians aged 10–12 years^(14)^. As measurable differences are believed to exist in terms of how older adolescents make decisions impacting their health, further research is necessary to understand which modifiable factors are linked with their MMA consumption. This will help to identify focus areas for targeted interventions to reduce disparities in MMA and Ca intake. Therefore, the purpose of this paper is to evaluate attitudinal, social and control factors that influence Canadian secondary school students’ MMA consumption and to explore associations through age and gender lenses.

## Methods

The research protocol, which included semi-structured interviews and photo-elicitation, received ethics clearance from the University of Waterloo Research Ethics Board. Prior to recruitment and data collection, the first author pilot-tested the data collection tool (see online supplementary material, Supplemental Table 1) on thirty undergraduate students at the University of Waterloo to ensure validity. Purposive sampling was then used to recruit adolescent participants from four groups of interest: junior (i.e. grades 9–10) girls, senior (i.e. grades 11–12) girls, junior boys and senior boys. Adolescent participants were recruited via digital and paper posters, advertising the research on websites (i.e. Reddit, Kijiji and Twitter) and community boards (i.e. grocery stores, community centres, YMCA, coffee shops and small shopping centres) throughout various municipalities within Ontario, Canada (i.e. Hamilton, Halton, Wellington and Kitchener-Waterloo), as well as snowball sampling. Interested individuals reached out to the study team by email and answered screener questions regarding their gender, grade of enrolment and municipality of residence. Gender was an open-ended response, determined by asking participants which gender they identified with most. Individuals were considered eligible if they were currently enrolled in a secondary school in one of the aforementioned municipalities. Recruitment proceeded iteratively throughout the research process until data saturation was reached (i.e. new data repeated what was expressed in previous data) during data collection^(16)^.

Informed, written consent and assent were obtained from parents/guardians and participants, respectively. Semi-structured interviews (*n* 28) were coupled with photo-elicitation, which was used to introduce the topic, elicit discussion and give participants autonomy during the interviews^(17)^. To begin each interview, participants were asked sociodemographic questions including whether they had a part-time job or participated in extracurricular activities, and the frequency and type of extracurricular involvement (see online supplementary material, Supplemental Table 1). Participants were then asked to discuss pictures of school vending machines and products for sale in the school and surrounding environments (e.g. ‘which product would you choose to purchase, and why?’) and probed to describe their decision-making process. Pictures depicted MMA as well as other beverages. Participants were asked questions to understand various environmental, attitudinal and social influences on their MMA consumption practices. A list of questions and probes used to guide the interviews is provided in online supplementary material, Supplemental Table 1. Participants were also asked to interpret demographic and behavioural characteristics associated with adolescents’ MMA consumption according to previous research by Butler *et al.* (e.g. ‘According to this study, girls in high school tend to consume fewer servings of MMA compared to boys. How would you explain this?’)^(1)^. All interviews were conducted by the first author over teleconferencing software or telephone and took place between February and July 2019. Each interview was between 30 and 65 min in length, audio-recorded, transcribed verbatim, and anonymised. All electronic files were stored on a computer with encrypted storage. Students received two volunteer hours as an incentive to participate.

Data analysis was informed by the Theory of Planned Behaviour (TPB), as a previous systematic review and meta-analysis has demonstrated its application to predict various dietary behaviours among youth^(18)^. The TPB is based on the presumption that people consider the consequences of their actions before making decisions^(19)^. Considering one of Riebl *et al.*’s findings – that adolescents’ intentions were a significant predictor of their eating behaviours – the authors considered adolescents to be rational decision-makers^(18)^. The TPB proposes that behaviour can be predicted by intentions to perform a behaviour, and that intentions are in turn determined by attitudes, subjective norms and perceived control over the behaviour^(19)^. According to the TPB, attitudes consist of an individual’s positive or negative evaluation of the behaviour^(19)^. Subjective norms are the social factors influencing behaviour and reflect the perceptions an individual holds about whether important others want them to perform or not to perform a particular behaviour^(19)^. Perceived control refers to the beliefs an individual holds about their ability to perform a behaviour^(19)^.

Thematic content analysis was carried out using NVivo 12 qualitative data analysis software, using both deductive and inductive approaches^(20)^. This allowed codes to be developed based on theoretical constructs in the TPB and the participants’ accounts. Inductive themes were generated by looking for repetition, dominant ideas, similarities and differences^(20)^. An initial coding scheme was created which was later consolidated before the codes were applied to interview transcripts. The coding scheme underwent several iterations before the codes were considered reliable. Intra-rater reliability was assessed to determine the consistency of codes applied by the first author over two separate coding sessions, and codes were 88 % consistent when applied 2 weeks apart. The final coding scheme was then applied to all transcripts by the lead author. Coding queries and matrix queries were conducted to explore the associations between groups of interest and attitudes, subjective norms, and perceived control codes. Gender and age lenses were applied by assigning participant attributes (e.g. gender and age) to each transcript and then charting data based on attributes and thematic codes to identify patterns.

## Results

### Participant characteristics

A total of twenty-eight students participated in interviews. Participants identified as boys (*n* 16) and girls (*n* 12) and were enrolled in grades 9–10 (*n* 19) and 11–12 (*n* 9) at the time of the study (see online supplementary material, Supplemental Table 2). Most participants identified as junior boys (*n* 12), followed by junior girls (*n* 7), senior girls (*n* 5) and senior boys (*n* 4). The results are organised based on the key concepts of the TPB – attitudes, subjective norms and perceived behavioural control – which, according to this theory, shape behavioural intentions and thus behaviour^(19)^.

### Attitudes

#### Health beliefs

Most participants (*n* 24) discussed health outcomes that they perceived to be associated with consuming MMA. Of these adolescents, sixteen students discussed predominantly favourable outcome evaluations, while six discussed predominantly unfavourable ones. The remaining two participants who expressed health beliefs perceived the outcomes of consuming MMA both favourably and unfavourably. Various subthemes emerged related to participants’ health attitudes, and the main subthemes will be discussed in the paragraphs that follow. It is important to note that participants’ attitudes are presented regardless of the availability of scientific evidence to support these attitudes.

Those who considered the outcomes of consuming MMA desirable expressed that consumption of MMA promotes good health, and that when MMA are consumed as part of a balanced diet, the consumer is more likely to meet their nutritional needs for health and physical fitness. Above all, they associated the behaviour of consuming MMA with health benefits such as strong bones, muscular strength and growth. Some explained that this was due to minerals like Ca and other important nutrients these foods contain. Many participants also shared the beliefs that consuming MMA leads to increased muscular strength and general growth (e.g. height):

*‘Milk just makes you stronger and you grow more […] It’s kind of like a stereotype again that milk helps kids grow, especially when they’re developing, like, around my age, in that it’s kind of like a joke that uh, if you drink a lot of milk you’ll grow taller’* (junior boy 13)

Enhanced muscular strength was perceived as desirable among boys. Many participants echoed these thoughts:

*‘[MMA] would be a good, good thing to consume for helping build muscle. And growing a bit. […] guys have a bit more muscle mass than women and we need to build it up a bit more.’* (junior boy 24)

A few participants also believed that ‘*chocolate milk helps you like recover [after physical activity]’* (senior boy 8).

On the other hand, participants who considered the health outcomes of consuming MMA undesirable largely discussed unwanted skin conditions, weight gain and diseases resulting from their consumption of these products. For instance, the belief that cows’ milk and cheese can trigger acne and eczema was commonly held among these participants. A junior boy described how this belief impacted his MMA consumption as he aged:

*‘Too much dairy products can cause, like acne and things like that. Since I’m in tenth grade now and I’m kind of going through that phase, I’ll tend to- like I’ll still have dairy but I just, I won’t go overboard, like if I was a kid.’* (junior boy 22)

The belief that MMA products are fattening and that their consumption can lead to weight gain was also discussed, and both subthemes were predominantly discussed among girls. Some felt that cheese was more fattening than other MMA products, and some felt that plant-based alternatives were less fattening. Similarly, some believed that consuming MMA leads to increased risk of disease, although these participants were uncertain of which diseases specifically:

*‘I’ve heard sometimes some diseases are linked to drinking cows’ milk? So that’s just that idea in my mind has made me sort of stay away and made me sort of think badly of cows’ milk compared to other milk alternatives’* (senior girl 28)

Some participants discussed both favourable and unfavourable evaluations of MMA, describing their experience of discerning facts from myths as difficult due to the spreading of misinformation.

#### Perceived enjoyment

Equal to health beliefs, participants discussed taste and the expected level of pleasure they associated with consuming MMA, comparing these attitudes to their attitudes towards other products. Of the participants interviewed, most held desirable attitudes towards the taste of MMA products while a minority exhibited undesirable or mixed attitudes. Many adolescents discussed taste preferences for flavoured chocolate milk over unflavoured milk. For example, one senior boy recalled a school milk programme with nostalgia, noting that chocolate milk was a favourite drink of his. Some participants also discussed taste as a deterrent from MMA consumption. While most expected to enjoy chocolate milk, most also expected not to enjoy other flavoured MMAs (e.g. fruity milks and milkshakes). Some participants described MMA that they perceived to contain large amounts of sugar as disgusting and off-putting. Overall, taste appeared to be a larger motivator among juniors and boys.

#### Environmental beliefs

In addition to health and enjoyment-related behavioural beliefs about consuming MMA, fourteen of the adolescent participants expressed environment-related beliefs. In general, adolescents’ perspectives on environmentally friendly choices influenced their preferences for MMA. Most of these participants attributed undesirable environmental outcomes to consuming cows’ milk and dairy products specifically. For example, when asked if she had a reason to avoid MMA, one senior girl exclaimed:

*‘This is something that I’ve definitely learned at school and like in health class and science class, just that when cows fart and they release like the methane I think it was […] that’s definitely a small factor when it comes to just drinking cows’ milk.’* (senior girl 28)

### Another participant expressed

*‘I’ve heard that cow milk is a lot more- it takes a larger toll on the environment than plant-based milks because there’s like the energy transfer- so the cows, I think you only get 10 % of the energy that the cows consume if that makes sense? […] But if you drink plant-based milks that cuts out the mailman of the cow, so you’re getting a lot more of the energy.’* (junior girl 23)

Indeed, participants’ belief that consuming MMA leads to unfavourable environmental outcomes was weaker for plant-based MMA compared with animal-based MMA. Many participants discussed the packaging of MMA products, attributing the consumption of MMA packaged in cartons to less environmental damage. Moreover, they considered this outcome desirable. While half of the participants’ perspectives on environmentally friendly choices influenced their attitudes towards MMA, the other half did not appear to hold such perspectives.

#### Other less prominent beliefs

Several other beliefs emerged from the interviews as minor themes. Animal welfare concerns were heard related to dairy production and farming. These participants felt that dairy cows did not have respectable living conditions. Additionally, a group of participants’ attitudes towards MMA were in part shaped by beliefs about unwanted additives to cows’ milk. For example, hormones and antibiotics were discussed.

### Subjective norms

Students also discussed social influences on their MMA consumption patterns. Parental influences were most frequently spoken of, followed by those of friends, coaches, teachers and health professionals. Over half of the participants (*n* 16) expressed normative beliefs regarding their parents, including the types of MMA to consume and avoid. For example, a senior girl conveyed her belief that her father wanted her to consume cows’ milk:

*‘Growing up I could tell my dad, he thought we should drink a lot of milk. It’s definitely gone down in the past two years, um, just because… I think he figures because we’ve stopped growing as much, we don’t need it as much. I dunno. But we have definitely gone back on the amounts of milk. But we used to drink, like, three glasses of milk a day. And it’s because he loves milk too, so like he used to drink it by the carton.’* (senior girl 20)

Indeed, parental influences on MMA consumption appeared to be stronger during early adolescence and weaker during late adolescence. Many participants echoed these thoughts:

*‘You’re not controlled by your parents as much in grade 12 so your parents aren’t there to tell you to drink more milk and you might not eat as much meals with them […] so your parents can’t really control how much milk you get anyway.’* (junior girl 23)

Weaker parental influences were associated with greater independence and independent mobility.

Besides holding normative beliefs about parents, some participants (*n* 6) expressed normative beliefs or motivations to comply with friends. For example, a friend’s belief about the healthiness of consuming cows’ milk for the perceived benefit of hair growth was discussed by a junior girl. Similarly, a senior boy considered his friend’s health belief regarding ‘unnatural additives’ to cows’ milk and considered their view that ‘alternatives like soy milk are better for you in the long run’ (senior boy 2).

Other social influences on participants’ MMA consumption were discussed less often. According to some participants who were involved in sports, coaches influenced their MMA consumption and so did dietitians and nutritionists who spoke to their sports team about healthy eating practices. Subjective norms including participants’ beliefs about whether doctors and health teachers wanted them to consume MMA were also discussed.

### Perceived behavioural control

Control beliefs also emerged from the interviews with high school students, including their perceptions about costs and the time required to prepare foods containing MMA. Accessibility, tolerability, willpower and circumstances (e.g. after sports) were additional factors discussed. Notably, fifteen participants discussed control beliefs regarding the cost of purchasing MMA, and they overwhelmingly viewed cost as a constraint on their MMA consumption. Relative to other drinks, participants viewed milk as expensive. Some participants noted that their consumption of MMA was limited to times when their favourite products went on sale or when their parents could afford the products: *‘Soy milk we sometimes get in the Chinese store […] but sometimes we don’t get it except if it’s on sale’* (junior boy 18). A junior girl explained: *‘Every once in a while I’ll have [goat cheese], once I can afford it- my mom can afford it’* (junior girl 3). In addition, seven participants commented that time was a factor that either facilitated or hindered their MMA consumption. Greater academic demands faced by senior students impacted the time these students had to prepare and consume foods, and the implications for MMA consumption appeared mixed. Some participants noted that they did not have time to eat breakfast, a meal they considered to typically include MMA. Others commented that MMA, such as milk on cereal, provided a quick and satisfying meal or snack when they were rushed. Moreover, some participants commented on the availability of MMA in their home and school affecting their ability to consume these products, and experiences were mixed.

Minor themes that emerged were the perceived level of tolerability that participants attributed to the act of either eating or drinking MMA and the self-efficacy for exerting willpower. For example, some students described resisting the temptation to choose less healthy drinks and making choices that align with their health beliefs about MMA.

## Discussion

This study provides an in-depth understanding of factors influencing Canadian secondary school students’ MMA consumption. By examining their attitudes towards MMA, prevailing subjective norms and perceived behavioural control, our findings help to contextualise demographic trends of low MMA and Ca intake among Canadians. Among the various factors associated with MMA consumption, the most cited were behavioural beliefs related to health, taste, and the environment, normative beliefs regarding parents, and control beliefs regarding cost. Previous studies corroborate the relevance of health beliefs, and attitudes more broadly, to dairy and Ca consumption among Canadians^(11–14)^. We add to this literature by identifying specific health beliefs held by adolescents so that these beliefs can be targeted in interventions. Previous research examining specific health beliefs held by Canadians is limited and constrained to the items in the questionnaire used^(13)^. However, our findings support those of Marcinow *et al.* regarding the perceived benefits of dairy consumption for strong bones^(13)^. Besides valuing their own health, adolescents predominantly valued the views of their parents when making decisions regarding their MMA consumption. Racey *et al.* also identified parents as predominant social influences on young Canadians’ dairy consumption^(14)^. Similarly, having family members who drink milk and parents’ rules concerning milk have been correlated with dairy consumption and Ca intake among undergraduate students^(15)^. While the present study found that cost was a predominant factor among control beliefs influencing MMA consumption, previous research offers inconsistent evidence on which control factors are most relevant to dairy and Ca consumption^(11–15)^. This variation may stem from differences in the age groups examined across the available studies and the considerable lifestyle changes that occur between adolescence and adulthood. According to research among youth aged 10–12 years, main control correlates of dairy consumption behaviour include availability, convenience, skipping meals, and habits or routines^(14)^. Cost has been correlated with adults’ Ca and dairy consumption however^(11–13)^. These findings regarding the importance of cost as a factor determining Canadians’ MMA intake make sense as youth tend to rely on their parents/guardians to purchase food. Our findings regarding the salience of cost are notable as previous research has not identified control factors which impact high school students’ MMA consumption, and findings from adult and child populations cannot be generalised to this age group. Interestingly, our findings suggest that environmental beliefs may also be impacting Canadians’ MMA consumption and particularly animal-based MMA. While environmental beliefs were not identified as main influences of MMA consumption in research examining attitudes towards MMA among adults^(11)^ and youth aged 10–12 years^(14)^, environmental beliefs emerged as a minor factor in Lacriox *et al.*’s research^(11)^. These findings are unsurprising given the hefty carbon footprint of dairy production, lapse in time since the aforementioned publications and increased public awareness about environmental issues^(21,22)^. Moreover, most of the less prominent findings reported in this study have also been reported by others^(11,14)^.

This research suggests some reasons for the gender disparities in Canadian adolescents’ MMA consumption. Notably, we found that both girls and boys held the behavioural belief that consuming MMA leads to increased muscular strength. However, this outcome was evaluated favourably predominantly among boys. Our findings suggest that adolescent girls may feel neutral or deterred from consuming MMA because of their belief that this behaviour leads to increased muscle mass. These gendered differences in health beliefs could contribute to teenage boys more readily meeting their nutrient requirements compared with girls^(1)^. Similarly, we found that adolescent girls discussed unfavourable health beliefs (i.e. skin conditions and weight gain) regarding MMA more often than adolescent boys. Similar to our findings regarding gender differences in health beliefs, Lacriox *et al.*’s findings reveal that environmental concerns related to MMA consumption shape women’s dietary behaviour but not men’s^(11)^. We did not find an association between gender and environmental attitudes towards MMA. However, these findings suggest that gendered beliefs may be an important explanatory factor regarding the observed gender differences in MMA and Ca intake^(1)^.

This research also suggests some reasons for the age disparities in Canadian adolescents’ MMA consumption. Above all, our findings suggest that lower prevalence of meeting recommendations for MMA in senior compared with junior years may in part be due to parental influences. Specifically, this research reveals that parental influences were the most cited among the social factors, and these influences appeared to be stronger for juniors compared with seniors. Taste appeared to be a larger motivator among juniors. To our knowledge, previous Canadian research has not examined behavioural factors to contextualise this age-related trend.

The insight gained from this research can be used to develop strategies for behaviour change interventions to improve Canadian adolescents’ MMA consumption. Given the salience of attitudinal factors among our results, strategies for behaviour change would benefit from addressing behavioural beliefs and outcome evaluations, according to the TPB^(19)^. This could include changing behavioural beliefs by dispelling myths about MMA (e.g. ‘many people think that consuming MMA leads to weight gain, but scientific evidence actually shows that consuming dairy is not related to excess fat accumulation during late childhood’^(23)^). Changing attitudes can also be achieved by changing the strength of an existing evaluation (e.g. ‘You know that consuming MMA helps to build and strengthen bones, but you may not know how critical nutrients in MMA are for bone establishment during this phase of your life’^(7)^). Our findings also support the use of gender-specific strategies for addressing behavioural beliefs. Moreover, to achieve behaviour change, the delivery of these messages is important. Given our finding that adolescents valued their parents’ views when making decisions about whether, how often and which MMA they consume, behaviour change interventions would likely be more effective if they included parents, or if parents communicated messages like the examples provided above to their children.

Dairy producers and the agricultural sector can continue to promote the uptake of their products among adolescent Canadians by addressing environmental concerns. This research supports the continued employment of policies and incentives regarding farming practices that counteract the greenhouse gas emissions generated by cattle. Educating Canadians on the steps being taken towards environmental sustainability is also critical, and dairy producers and the agricultural sector are encouraged to continue these efforts. The use of specific feed mixes (e.g. incorporating essential oil blends) in dairy farming are one strategy for reducing methane emissions, while positively affecting milk yields, that has been supported by literature^(24)^. Another strategy for improving the sustainability of farming practices is the use of rotational grazing, which are pasture management practices designed to increase the quality and quantity of forage^(25)^. Producers can also continue partnering with other organisations and stakeholders to promote sustainability. Finally, supporting research and development of more biodegradable solutions to MMA packaging and ceasing the use of disposable plastics is advised.

Subsidies, incentives and programmes to promote availability of low-cost MMA are recommended. Policies that promote the availability and accessibility of low-cost MMA would help to reduce barriers that Canadian adolescents face in meeting their nutrition requirements.

This study has some limitations. Overall, research participants held favourable attitudes towards MMA, and this may be indicative of volunteer bias, as those who were interested and enjoyed consuming MMA volunteered to participate. As such, this study may not have fully captured the perspectives of adolescents who do not hold favourable views of MMA. Beyond gender, education level, school board and census division, additional demographic attributes of participants (e.g. ethnicity) were not obtained. This makes it difficult to contextualise other observed disparities in MMA consumption among adolescents. Moreover, this research did not incorporate the views of non-binary individuals; more research in this area is needed.

## Conclusion

This research contextualises recent trends in Canadians’ MMA consumption, including gender and age disparities. Our use of the photo-elicitation exercise allowed for fruitful conversations later in the interview as participants appeared to gain comfort during the exercise. They spoke openly about the factors that influence their MMA consumption, including attitudes, subjective norms and perceived control. The practical implications of these findings highlighted here are useful for public health promoters, dairy farmers, other MMA producers and policymakers.

## Supporting information

Thompson et al. supplementary material Thompson et al. supplementary material

## Supplementary material

For supplementary material accompanying this paper visit https://doi.org/10.1017/S1368980024000661.
