# Supplementary material for: Attitudes, subjective norms and perceived behavioural control factors influencing Canadian secondary school students’ milk and milk alternatives consumption
Source: Public Health Nutr. 2024 Mar 8;27(1):e92. doi: 10.1017/S1368980024000661 (PMC10966828; doi:10.1017/S1368980024000661)
Supplement: Thompson et al. supplementary material [file S1368980024000661sup001.docx]

**Table 1.** Semi-structured interview guide

| Interview questions | Probes |
| --- | --- |
| Sociodemographic questions |  |
| Who talks to you about food including MMA? | What do they say? |
| If you could choose one of these products from the vending machine to purchase, which would you choose and why? | Can you describe your decision-making process?  If you would not consume any of these products, why not? |
| What do you know about the way these products are made? | Would this impact whether and which MMA products you eat? |
| Within the MMA category, which foods and drinks, if any, do you choose to eat most often? | Why do you choose/not choose these foods? |
| Where are you when you tend to consume these products? | For example, are you at home, school, a friend’s house? |
| Do you believe there is any risk for you or for others in consuming any of the MMA? | If health reasons are mentioned: Are there any other ways that you think about any of the MMA related to health?  Where do you typically get this information from? |
| Do you have any reasons to avoid any of the MMA? |  |
| According to a research project with high school students (Butler et al., 2020), girls tended to consume fewer servings of MMA compared to boys.  Does this finding make sense to you, and can you explain why or why not based on your own experience? |  |
| A second finding from this research (Butler et al., 2020) was that students tended to meet MMA guidelines less often with each increase in grade (i.e., from grade 9 to grade 12).  Does this finding make sense to you, and can you explain why or why not based on your own experience? |  |
| A third finding from this research (Butler et al., 2020) was that students who tend to meet other nutrition recommendations and have healthy body weights also tend to meet MMA guidelines.  Does this finding make sense to you, and can you explain why or why not based on your own experience? | What or who influences you (or other students) to have healthy habits? |
| Ending questions |  |

**Table 2.** Sample characteristics

| Sociodemographic  Factors |  | Number of  Participants (%) |
| --- | --- | --- |
| Census Division | Regional Municipality of Waterloo  City of Hamilton  Regional Municipality of Halton  Wellington County  Middlesex County  Other | 8 (29)  7 (25)  6 (21)  3 (11)  3 (11)  1 (4) |
| Education Level | Grade 9-10  Grade 11-12 | 19 (68)  9 (32) |
| Gender | Boys  Girls | 16 (57)  12 (43) |
| School Board | English Public  English Catholic  Homeschooled or alternative  Private | 20 (71)  4 (14)  2 (7)  2 (7) |
| Type of extracurricular involvement | Sports  Art  Academic  Community Service  Other | 14 (45)  6 (19)  5 (16)  4 (13)  2 (6) |
| Frequency of extracurricular involvement | 1-2 times per week  3 or more times per week  Monthly or seasonally  Never or rarely | 11 (39)  7 (25)  5 (18)  5 (18) |
| Part time job | No  Yes | 22 (79)  6 (21) |
| Total |  | **28 (100)** |
